# PolyA-miner: accurate assessment of differential alternative poly-adenylation from 3′Seq data using vector projections and non-negative matrix factorization

**DOI:** 10.1093/nar/gkaa398

**Published:** 2020-05-28

**Authors:** Hari Krishna Yalamanchili, Callison E Alcott, Ping Ji, Eric J Wagner, Huda Y Zoghbi, Zhandong Liu

**Affiliations:** Department of Molecular and Human Genetics, Baylor College of Medicine, Houston, TX 77030, USA; Jan and Dan Duncan Neurological Research Institute, Texas Children's Hospital, Houston, TX 77030, USA; Jan and Dan Duncan Neurological Research Institute, Texas Children's Hospital, Houston, TX 77030, USA; Program in Developmental Biology, Baylor College of Medicine, Houston, TX 77030, USA; Medical Scientist Training Program, Baylor College of Medicine, Houston, TX 77030, USA; Department of Biochemistry & Molecular Biology, University of Texas Medical Branch, Galveston, TX, 77555, USA; Department of Biochemistry & Molecular Biology, University of Texas Medical Branch, Galveston, TX, 77555, USA; Department of Molecular and Human Genetics, Baylor College of Medicine, Houston, TX 77030, USA; Jan and Dan Duncan Neurological Research Institute, Texas Children's Hospital, Houston, TX 77030, USA; Howard Hughes Medical Institute, Houston, TX 77030, USA; Department of Pediatrics, Baylor College of Medicine, Houston, TX 77030, USA; Department of Neuroscience, Baylor College of Medicine, Houston, TX 77030, USA; Jan and Dan Duncan Neurological Research Institute, Texas Children's Hospital, Houston, TX 77030, USA; Department of Pediatrics, Baylor College of Medicine, Houston, TX 77030, USA

## Abstract

Almost 70% of human genes undergo alternative polyadenylation (APA) and generate mRNA transcripts with varying lengths, typically of the 3′ untranslated regions (UTR). APA plays an important role in development and cellular differentiation, and its dysregulation can cause neuropsychiatric diseases and increase cancer severity. Increasing awareness of APA’s role in human health and disease has propelled the development of several 3′ sequencing (3′Seq) techniques that allow for precise identification of APA sites. However, despite the recent data explosion, there are no robust computational tools that are precisely designed to analyze 3′Seq data. Analytical approaches that have been used to analyze these data predominantly use proximal to distal usage. With about 50% of human genes having more than two APA isoforms, current methods fail to capture the entirety of APA changes and do not account for non-proximal to non-distal changes. Addressing these key challenges, this study demonstrates PolyA-miner, an algorithm to accurately detect and assess differential alternative polyadenylation specifically from 3′Seq data. Genes are abstracted as APA matrices, and differential APA usage is inferred using iterative consensus non-negative matrix factorization (NMF) based clustering. PolyA-miner accounts for all non-proximal to non-distal APA switches using vector projections and reflects precise gene-level 3′UTR changes. It can also effectively identify novel APA sites that are otherwise undetected when using reference-based approaches. Evaluation on multiple datasets—first-generation MicroArray Quality Control (MAQC) brain and Universal Human Reference (UHR) PolyA-seq data, recent glioblastoma cell line *NUDT21* knockdown Poly(A)-ClickSeq (PAC-seq) data, and our own mouse hippocampal and human stem cell-derived neuron PAC-seq data—strongly supports the value and protocol-independent applicability of PolyA-miner. Strikingly, in the glioblastoma cell line data, PolyA-miner identified more than twice the number of genes with APA changes than initially reported. With the emerging importance of APA in human development and disease, PolyA-miner can significantly improve data analysis and help decode the underlying APA dynamics.

## INTRODUCTION

Advances in sequencing techniques have improved our understanding of the transcriptome and unraveled new mechanisms of complex diseases. However, several critical aspects of transcriptome diversity are underexplored. In the mRNA maturation process, the 3′end of precursor mRNA (pre-mRNA) is cleaved and a poly(A) sequence is added. In eukaryotes, all pre-mRNA molecules except histones undergo polyadenylation (1). In humans, ∼70% of genes undergo alternative polyadenylation (APA), where they can be cleaved at different sites on the 3′ end, generating mRNA transcripts of varying lengths (2). ∼50% of human genes have three or more polyadenylation sites (3). Multiple studies have demonstrated the pivotal role of alternative polyadenylation in key biological processes including gene regulation (2), mRNA localization (4), cell proliferation (5), differentiation (6), and senescence (7). The importance of alternative polyadenylation is also demonstrated in the development and prognosis of various oncological, neurological, immunological, and endocrinal diseases (8).

**Figure 1.**
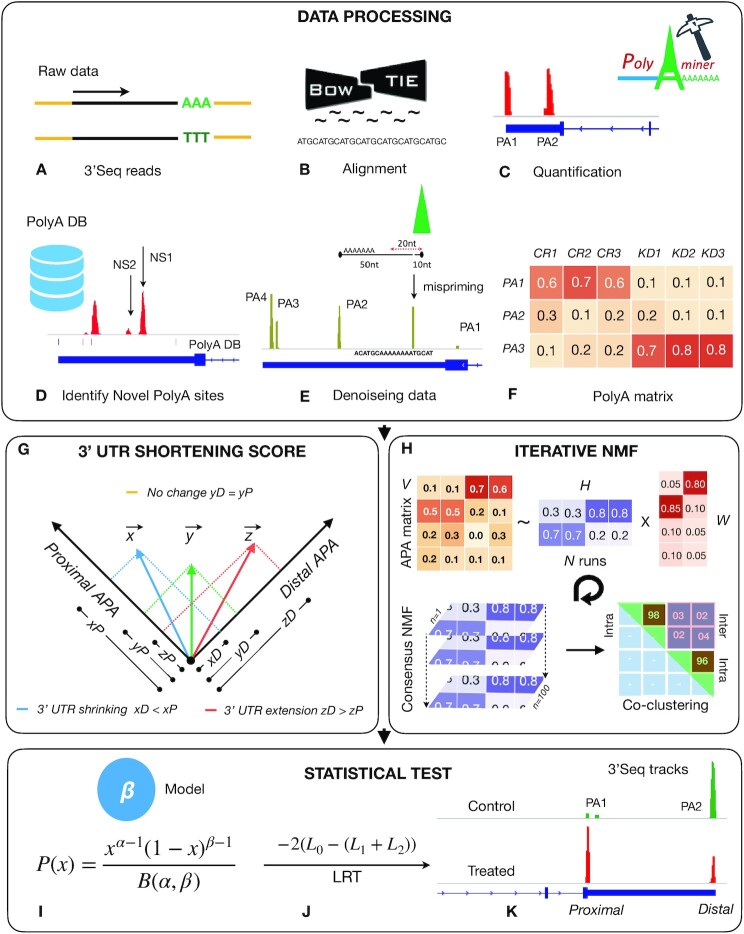
Illustration of PolyA-miner pipeline: (**A**) Raw 3′Seq reads. (**B**) Alignment. (**C**) Quantification of APA peaks: PA1 and PA2 are polyadenylation sites 1 and 2 respectively. (**D**) Identifying novel APA sites: NS1 and NS2 are novel polyadenylation sites that are not reported in PolyA_DB. (**E**) Denoising data: cleaning misprimed sites and noisy APA peaks. (**F**) Normalized APA matrix: each row is a polyadenylation site and columns are the read proportions in respective CR (control) and KD knockdown replicates. (**G**) Vector projection module to compute differential APA magnitude. (**H**) iterative consensus non-negative matrix factorization (NMF) module. (**I**) Modeling co-clustering frequencies. (**J**) Goodness of fit test of cluster membership over a null model. (**K**) Tracks showing detected APA changes.

Expressed sequence tags (EST) (9) were initially used to map polyadenylation sites. Later on, microarray and paired-end ditag (PET) approaches were used to detect global APA changes (10). But it was the next-generation sequencing (NGS) technology that started to transform APA analysis. RNA-Seq offers single-base resolution and a wider detection range in identifying novel genes, splice forms and non-coding transcripts. However, because of the huge intrinsic variation of read coverage at the 3′ end, precise mapping and quantification of polyadenylation sites is not possible. When looking at mapped reads, shorter 3′ UTR transcripts are undetectable unless they are expressed at dramatically higher levels than the longer isoforms. The increasing significance of APA in disease coupled with the limitations of traditional RNA-Seq propelled the development of several 3′RNA-seq techniques specifically designed to identify the mRNA cleavage and polyadenylation sites. These methods include 3′Seq, polyadenylation sequencing (PA-seq) and poly(A) site sequencing (PAS-seq), all of which use oligo(dT) primer based reverse transcription to capture the 3′ end of mRNA (Figure 1a). However, they all suffer from significant poor base-calling quality and mispriming, where the poly(dT) primer that is intended to bind the poly(A) tail instead binds a sequence of genomic adenines (11). Techniques like poly(A)-test RNA-sequencing (PAT-seq) and poly(A)-position profiling (3P-seq) try to minimize mispriming by adding adapters prior to primer annealing, but require complex RNA manipulation steps and perform poorly in quantification (3). Limiting factors like poly(A) enrichment and 3′ linker ligation steps are bypassed by Poly(A)-ClickSeq (PAC-seq) using click-chemistry (12), which can also be used for differential expression analysis (13)

With the explosion of emerging technologies, we need new analytical methods. In spite of increasing significance of APA and 3′ sequencing (3′Seq) techniques, there are no computational tools that are designed precisely for 3′Seq data. Several studies have analyzed conventional RNA-seq data to infer alternative polyadenylation changes. DaPars (14) uses a fisher exact test on proximal to distal APA site differences. Similarly, QAPA (15) uses DEXseq (16) and TAPAS (17) uses a change point strategy to infer APA changes from regular RNA-seq. However, none of them are 3′Seq specific and do not identify and account for all APA isoforms. Although previous studies have revealed the core insights from 3′seq data, their analyses have largely been incomplete. Those approaches typically either relied on existing poly-A annotations and ignored novel APA sites or were limited to proximal and distal polyadenylation sites (18) and ignored APA changes involving intermediate poly(A) sites (Supplementary Figure S1). Enrichment of proximal or distal polyadenylation sites, commonly referred to as proximal to distal usage (PDU) or distal to primal usage (DPU), are computed to infer gene level APA changes (18,19). With almost 50% of genes having more than two polyadenylation sites, gene level APA changes are better comprehended by accounting for all polyadenylation sites. However, none of the existing approaches abstract all polyadenylation sites in quantifying gene level APA changes. The absolute lack of 3′Seq specific approaches strongly advocate the need for new computational methods to accurately assimilate the merits of 3′Seq data.

Here, we propose PolyA-miner, a novel *de novo* differential alternative polyadenylation detection algorithm based on non-negative matrix factorization (NMF) (20) and vector projections. NMF is popularly used for feature extraction in image processing (21). It is also successfully used to elucidate (factorize) gene expression patterns (22). It provides an intuitive interpretation of the factorization and parts-based, local representation in contrast to other well-known methods (23). The current context of alternative polyadenylation (APA) changes is analogues to clustering gene expression patterns where polyadenylation sites are represented as rows and samples as columns. NMF is a good fit when the attributes are ambiguous or have weak predictability (24). This is advantageous to account for all polyadenylation (polyA) sites with no single dominant polyA site. PolyA-miner tests how well the unsupervised consensus clustering of an APA matrix agrees with the ground truth (class labels). Co-clustering frequencies from iterative NMF are modeled as a beta distribution and the statistical significance of APA change is evaluated by the goodness-of-fit of the consensus clustering over a null model. Differential APA magnitude is computed as the difference of APA vector projections on to the most distal APA site in an *n-*dimensional vector space, where *n* is the number of APA sites. The methodical flow of PolyA-miner is illustrated in Figure 1. PolyA-miner is evaluated with both the first generation MAQC brain and UHR PolyA-seq data (3), on recent Glioblastoma cell line *NUDT21* knock down PAC-seq (3′Seq) data (19), and our own mouse hippocampal and human stem cell-derived neuron PAC-seq data. A detailed description of the proposed approach is given in the following Materials and Methods section.

## MATERIALS AND METHODS

### Processing raw reads

Irrespective of sequencing protocol nucleotide composition is biased at the beginning of reads due to random hexamer priming while amplifying cDNA (25). To improve the mappability (26), the first six nucleotides (12) and adapter contamination is filtered out using fastp (27). To minimize ambiguous alignment, reads <40 bp are also filtered out. Raw reads are then mapped to the reference genome of origin using bowtie2 (28). Alignment files in *‘sam’* format are converted to *‘bam’*, sorted and indexed using samtools (29).

### De novo extraction of alternative polyadenylation sites

All potential sample-wise poly adenylation (polyA) sites are extracted from alignment files as per base coverage features/peaks using the genomecov module in bedtools (Figure 1C). A comprehensive library of polyadenylation sites is computed by pooling all sample-wise feature files. To account for any intrinsic limitations in sequencing protocols, polyA sites that overlap or are within a minimal distance *md* are merged (Supplementary Figure S2e). Parameter *md* can be adjusted based on polyA resolution supported by the respective sequencing protocol used. Since 3′ sequencing methods use poly(dT) primers—to bind the mRNA poly(A) tail—they can also bind stretches of adenines within the body of the mRNA, resulting in sequencing reads that do not align with the cleavage site (30). We call these misprimed reads. Such misprimed sites are computationally filtered by exploring the downstream base composition (31). Typically, sites with greater than 15nt out of 20 nucleotides (75%) are considered false positives (12). However, here we took a more conservative approach: each mapped polyA site is extended towards the 3′ end by a mispriming distance *mpd* and scanned for a genomic PolyA feature. Sites with >65% of adenines in a sliding window of 20 bp are filtered out as shown in Figure 1E. Sites within 50 bp of an annotated cleavage site (32) are considered accurate regardless of the percentage of adjacent adenines.

### Mapping, denoising and normalizing APA counts

After filtering out misprimed sites, resulting polyA sites are mapped to their respective genes. Often times novel polyA sites fall beyond the annotated gene boundary. Because the longest known 3′UTR is 16 kilobases (kb) (33), APA sites are mapped to genes if they are within this distance of their respective transcriptional end site (TES) and do not overlap with any other gene (illustrated in Supplementary Figure S2a). Sample-wise polyA site counts are computed as the total number of reads mapped to the respective polyA site intervals using featureCounts (34). Each gene is conceptualized as a matrix with APA sites as rows and sample replicates as columns. To restrict the untoward effect of sequencing noise, polyA sites failing the pOverA function are filtered out. This function evaluates whether the proportion of replicates larger than A (reads) exceeds p with a minimum of *M* reads per site in at least one test group (illustrated in Supplementary Figure S2b). APA matrix is further pruned by filtering out the sites that fall short of a minimum proportion *mp* of total reads mapped to the respective gene in both the conditions (Supplementary Figure S2c). To constrain the APA changes due to non-expressed genes, genes with less than a minimum expression count *me* in either of the conditions are filtered out (Supplementary Figure S2d).

### Iterative Consensus non-negative matrix factorization (NMF)

PolyA-miner uses iterative consensus clustering to detect alternative polyadenylation changes (Figure 1H). Typically, clustering techniques are used to group samples or data points. However, in the current context of differential alternative polyadenylation, we have *a priori* information of sample clustering, i.e., a specified set of control and treated samples. The key here is to test the agreement between the clustering consensus of an APA matrix and the ground truth of a priori sample labels, which is essentially a factorization problem. Non-negative matrix factorization (NMF) is an unsupervised clustering paradigm that has previously been demonstrated for multivariate decomposition (35). Given an *m* x *n* dimensional non-negative APA matrix *V*, where *m* is the number of APA sites and *n* is the number of samples, we factorize *V* into an *n* × *k* matrix *W* and a *k* × *m* matrix *H* such that: *V_m_*_×_*_n_ ≈ W_n_*_×_*_k_ H_k_*_×_*_m_*, where *k* is the number of clusters. In our example, we have two clusters: control and treated (Supplementary Figure S3a). Factorization is approximated by minimizing the cost function (Supplementary Figure S3a–c):

$$\begin{equation*}F(W,H)\, = \,\left\| {V - WH} \right\|_F^2\end{equation*}$$

We used an efficient Coordinate Descent method (36) to solve *W* and *H*. Cluster membership is inferred from the *H* matrix, a sample is assigned to a cluster *i* if *H_i,m_ > H_j,m_*. detailed account of NMF is described elsewhere (37). Initialization can introduce potential bias in NMF (38). To minimize this, we execute NMF iteratively (Supplementary Figure S3d) and infer a robust dichotomization (control versus treated). An *n* x *n* co-clustering consensus matrix *CM* is computed (Supplementary Figure S3e) from the independent iterative NMF runs, where *n* is the number of samples.

$$\begin{eqnarray*} {C{M_{i,j}}} = \left\{\begin{matrix} 1\quad if\;i = j\\[4pt] {{C_{ij|k}}/ni}\\[4pt] 0\quad if\;i > j \end{matrix}\right. \end{eqnarray*}$$

*CM_i,j_* is the co-clustering frequency of samples *i* and *j*. *C_ij|k_* is the number of time sample *i* and *j* are assigned to the *a priori* cluster *k* and *ni* is the number of NMF iterations.

### Modeling beta distribution and likelihood-ratio (LRT) test

Co-clustering frequencies from iterative NMF are modeled using a beta distribution (Supplementary Figure S3f). Beta distribution is widely used to model outcomes that are constrained within a defined interval [0 to 1] and two parameters α, β, controlling the distribution shape. The probability density function (pdf) of a random variable *X* following beta distribution, *X∼Beta(α,β)* is given by:

$$\begin{eqnarray*} P(x) &=& \frac{{{x^{\alpha - 1}}{{\left( {1 - x} \right)}^{\beta - 1}}}}{{B\left( {\alpha ,\beta } \right)}}\\ &=&\left\{\begin{array}{ll} \frac{{{\rm{\Gamma }}\left( {\alpha + \beta } \right)}}{{{\rm{\Gamma }}\left( \alpha \right){\rm{\;\Gamma }}\left( \beta \right)}}{{\left( {1 - x} \right)}^{\beta - 1}}\;{x^{\alpha - 1}} & \quad if\;0\; \le x \le 1\\[4pt] 0 & \quad else \end{array}\right. \end{eqnarray*}$$

where *α* > 0, *β* > 0 and ${\rm{\Gamma \;}}( a ) = \mathop \smallint \nolimits_0^{ + \infty } {x^{a - 1}}\;{e^{ - x}}\;dx$ The mean and variance of a beta distribution are given by:

$$\begin{equation*}\bar x \approx \,\frac{\alpha }{{\alpha + \beta }};\,\,\,\,\,{s^2} \approx \frac{{\alpha \beta }}{{{{(\alpha + \beta )}^2}(\alpha + \beta + 1)}}\end{equation*}$$

We model co-clustering frequencies from CM matrix as a beta distribution. The parameters *α,β* are estimated using the moments method.

$$\begin{equation*}\beta = \frac{{\alpha (1 - \bar x)}}{{\bar x}};\,\,\,\,\alpha = \bar x\left[ {\frac{{\bar x(1 - \bar x)}}{{{s^2}}} - 1} \right]\end{equation*}$$

The log likelihood function of a beta model measure how well they fit the underlying data and is given by:

$$\begin{equation*}L\left( {\alpha ,\beta } \right) = \mathop \sum \limits_{i = 1}^N {\rm{log}}\left( {\frac{{{\rm{\Gamma }}\left( {\alpha + \beta } \right)}}{{{\rm{\Gamma }}\left( \alpha \right){\rm{\;\Gamma }}\left( \beta \right)}}\;{x^{\alpha - 1}}{{\left( {1 - x} \right)}^{\beta - 1}}} \right)\end{equation*}$$

Three sets of parameters and respective likelihoods are estimated for intra-group *L*(*α*_1_,*β*_1_) and inter-group *L*(*α*_2_,*β*_2_) and null *L*(*α*_0_*,β*_0_). Intra-group likelihood is modelled on the co-clustering frequencies of samples with the same *a priori* condition (control or treated), the inter-group likelihood is modeled on the co-clustering frequencies of samples from different conditions (control and treated), and a null distribution is modelled on the whole *CM* matrix. Differential polyadenylation is tested by evaluating the goodness-of-fit of the respective intra-group and inter-group co-clustering frequencies over the null model. A likelihood ratio test statistic is computed as the ratio of a simpler null model *s* to a complex alternative model *g*.

$$\begin{eqnarray*} {{\rm LRT}} &=& - 2{\log_{\rm e}}({L_{\rm s}}/{L_g})\\ &=& - 2{\log_{\rm e}}({L_s}/{L_{\rm g}})\\ &=& - 2({L_0} - \left( {{L_1} + {L_2}} \right)) \end{eqnarray*}$$

where L1, L2 and L0 are intra group, inter group and null model log likelihoods. LRT statistic is approximated as a χ^2^ distribution with 2 degrees of freedom and the *P* value is computed accordingly (Supplementary Figure S3g).

### Magnitude of alternative polyadenylation (APA) change

Genes undergoing polyadenylation changes often have more than two APA sites (3) and the changes are not always at the most distal and most proximal cleavage sites. The ideal magnitude metric should reflect changes at all APA sites that affect 3′UTR length. Vector projection is a good means to quantify a multi-dimensional variable (w.r.t a reference). An intuitive way to understand a projection of a vector *u* on vector *v* is the shadow of vector *u* on vector *v* (illustrated in in Figure 4A).

$$\begin{equation*}\left\| {pro{j_v}\vec u} \right\| = \left\| {\frac{{\vec u{\rm{\;}}{{\rm{\;}}^.}{\rm{\;}}\vec v}}{{{{\left\| {{{\vec v}^2}} \right\|}^2}}}{\rm{\;}}\vec v} \right\|{\rm{\;}} = \frac{{\left\| {\vec u{\rm{\;}}{{\rm{\;}}^.}{\rm{\;}}\vec v} \right\|}}{{\left\| {{{\vec v}^2}} \right\|}}{\rm{\;\;\;}}\left\| {{{\vec v}^2}} \right\| = \frac{{\left| {\vec u{\rm{\;}}{{\rm{\;}}^.}{\rm{\;}}\vec v} \right|}}{{\left\| {{{\vec v}^2}} \right\|}}\end{equation*}$$

Projection of the most proximal or distal APA site effectively resonate 3′UTR shrinking or lengthening respectively. Control and treated APA matrices are vectorized by a row mean operation and the magnitude of APA change is computed as the difference in projections of respective APA vectors on to the most proximal APA site in an *n* dimensional vector space, where *n* is the number of APA sites. Genes with higher distal projections in controls over treated are predicted as 3′UTR shortening and vice versa as illustrated in Figure 4B.

## RESULTS AND DISCUSSION

### Plethora of misprimed and noisy APA sites are filtered by PolyA-miner

**Figure 2.**
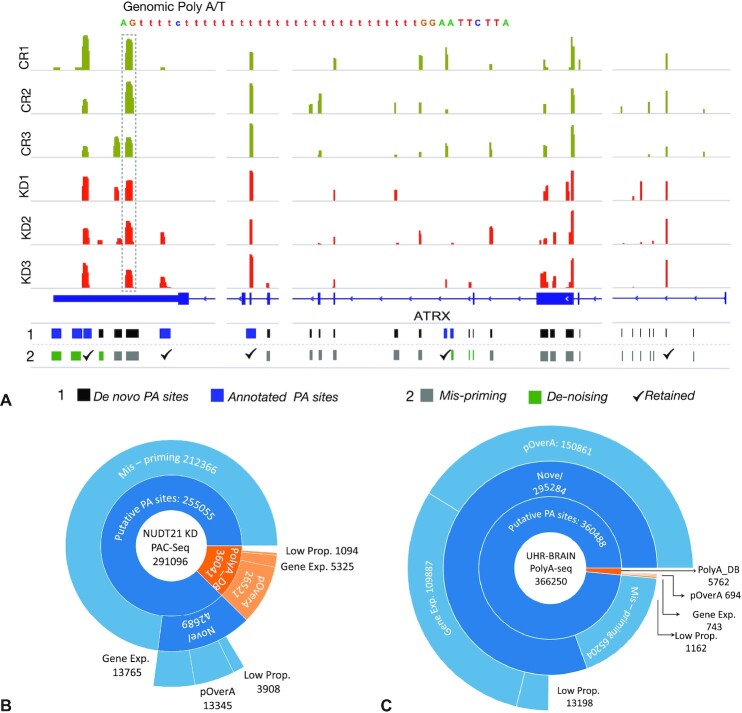
(**A**) Illustration of mispriming and other noise levels in the gene ATRX from *NUDT21* KD PAC-Seq data. Lane 1 shows all extracted polyA sites from the zoomed in region: Shown in blue are annotated sites from PolyA_DB and shown in black are putative sites. Highlighted in box is a representative misprimed site. Lane 2 shows polyA site that are flagged by different Poly-miner filters: Shown in grey are misprimed sites and shown in green are flagged by de-noising filters. Marked with tick (✓) are the retained polyA to test for APA changes. Distribution of the filtered putative and annotated APA sites by respective de-noising filters in (**B**) *NUDT21* KD PAC-Seq data and (**C**) MAQC brain-UHR PolyA-seq data.

A majority of 3′Sequencing datasets suffer from misidentification of spurious and noisy APA sites due to oligo (dT) internal priming of polyadenine stretches within the body of mRNA rather than the poly(A) tail. To evaluate the ability of PolyA-miner to filter misprimed sequencing reads, we applied our method to the Glioblastoma (GBM) LN229 cell line *NUDT21* knock down (KD) PAC-Seq data (SRP172550). This dataset is first reported in Chu *et al.* (2019) and was generated to help elucidate the contribution of *NUDT21* dependent APA regulation in GBM progression. We examined several genes that showed high levels of mispriming. Seven alternative polyadenylation (APA) sites are annotated for the gene ATRX in PolyA_DB, a database of APA sites backed by sequencing evidence (32). PolyA-miner detected 30 APA sites in the zoomed-in region shown for *ATRX* (Figure 2A) including 23 new putative novel sites. Out of the 23 putative sites PolyA-miner filtered out 19 putative sites with >65% of genomic adenines in a sliding window of 20 bp as described in the methods. A representative misprimed site with genomic poly thymine stretch (in negative sense strand) is annotated in Figure 2A. Furthermore, three of the four putative sites retained after mispriming filter and three of the seven annotated APA sites are filtered out by pOverA and other de-noising filters (Figure 2A). Similarly, we detected five out of six and three out of four misprimed putative sites in the genes *PAK2* and *IDS* (Supplementary Figure S2e and d). In addition, out of the seven PolyA_DB annotated sites in IDS, three are dropped by denoising filters. At the transcriptome level, 212 366 of the total 255 055 identified putative APA sites are potentially misprimed (Figure 2B), a remarkable 83%. De-noising pOverA and proportion filters dropped 17 253 novel and 27 615 annotated APA sites that are inconsistent across respective replicates. Furthermore, the gene expression filter dropped 13 765 novel and 5325 annotated sites to control for non-expressed genes in either of the conditions.

To thoroughly understand the noise levels in 3′ UTR sequencing data by ruling out any technical or species bias, we generated our own 3′Seq data from wild type mouse hippocampi and human stem cell-derived neurons (Supplementary methods). Both the mouse and human data had significantly high levels of mispriming and noise (Supplementary Figure S4a and b), similar to previous *NUDT21* knock down PAC-seq data (Figure 2B). These observations demonstrate the high levels of false positive polyA site identification inherent to 3′ sequencing and substantiate the merit of PolyA-miner to properly mine and interpret 3′ sequencing data. Our data is uploaded to GEO and will be a useful resource for investigating various technical aspects of 3′Seq data and better understanding the transcriptome of those tissues.

### More than twice the number of APA changes are identified in *NUDT21* knock down PAC-seq data than initially reported

**Figure 3.**
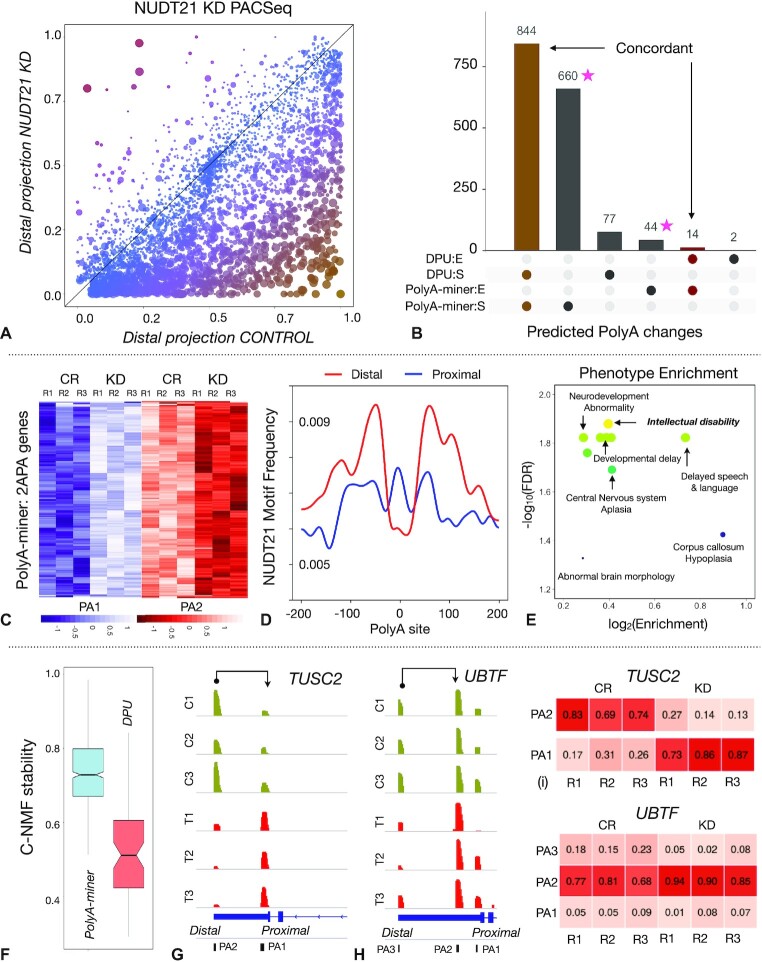
(**A**) PolyA-miner reveals extensive 3′UTR shortening in LN299 Glioblastoma *NUDT21* knock down data: Size of the marker indicate statistical significance. Color gradient (red to orange) indicate 3′UTR length long to short. (**B**) Overlap and discordance of PolyA-miner and DPU predictions. (**C**) APA heatmap of genes with two polyadenylations that are predicted to be 3′UTR shortening only by PolyA-miner. (**D**) NUDT21 motif frequency in genes that are predicted to be 3′UTR shortening by PolyA-miner. (**E**) Phenotype analysis of PolyA-miner results. (**F**) Clustering stability of PolyA-miner and DPU only predictions. (**G** and **H**) Tracks showing 3′UTR shortening identified by both PolyA-miner and DPU, and only PolyA-miner. (**I** and **J**) APA proportion matrices of the genes shown in (G) and (H) respectively.

APA vector projections are more proximal (less distal) in *NUDT21* KD samples than controls, suggesting global 3′UTR shortening (Figure 3A). This observation is in agreement with previously published analyses (19). However, the study reported only 695 genes with APA changes. PolyA-miner identified a striking 1562 genes with APA changes, revealing extensive APA dynamics (Figure 3A and B). To evaluate the methodological merit in an un-biased setup, consistent mispriming and denoising filters are applied to both the distal to proximal usage (DPU) and PolyA-miner. Annotated polyA sites from polyA_DB are used to control for novel APA site discovery advantage of PolyA-miner. As the executable code is not available for a direct comparison of the DPU approach with PolyA-miner, we repeated the analysis (supplementary methods). The DPU approach detected 921 genes with 3′UTR shortening and 16 with elongation (Adjusted *P* value ≤ 0.05). Among them, 844 (92%) and 14 were also detected by PolyA-miner with a total of 1504 3′UTR shortening and 58 3′UTR elongation changes (Figure 3B), including *VMA21*, a well-established positive control gene reported in previous *NUDT21* KD studies. The complete list of detected APA changes by PolyA-miner and DPU approach are given in Supplementary Tables S1 and S2 respectively. These data show that PolyA-miner replicates the results of previous methods and expands them.

### Concordant APA patterns strongly substantiate novel PolyA-miner predictions

PolyA-miner predicted 660 3′UTR shortened genes that are missed by DPU (Figure 3B). These hits are grouped by the number of APA sites in the visual illustration. The heatmap of the genes with two APA sites suggest conclusive 3′UTR shrinking (Figure 3C). Striking dark blue (low) to light blue (high) and contrariwise light red (high) to dark red (low) patterns are observed at the proximal and distal polyadenylation sites between control and *NUDT21* KD samples respectively. This high proximal and low distal pattern in *NUDT21* KD samples illustrate 3′UTR shortening. Genes with three APA sites also show similar apparent differential usage patterns at the proximal (blue: low to high) and distal (green: high to low) sites (Supplementary Figure S5). Together, two and three APA site genes constitute 466 (70%) of PolyA-miner only predictions. Heatmaps of 4 and 5 APA genes are also shown in Supplementary Figure S5.

Additionally, to further substantiates PolyA-miner predictions, we explored the distribution of the NUDT21 binding motif in the predicted targets. Earlier studies showed that NUDT21 binds to UGUA motif and reported global 3′UTR shrinking with a significant enrichment of the UGUA motifs near the distal polyA sites compared to the proximal polyA sites in Nudt21 knockdown models (18). In agreement with previous reports, we found an enrichment of the UGUA binding motif frequency upstream of the distal cleavage site in the genes that showed significant 3′ UTR shortening after *NUDT21* loss (Figure 3D). In contrast, no difference in UGUA motif distribution was found between proximal and distal polyA sites of the genes with no APA changes or lengthened (Supplementary Figure S6). This observation supports the model that NUDT21 is directed to distal sites to facilitate polyadenylation and thereby corroborate novel PolyA-miner APA predictions.

We next examined the clustering stability scores (described in supplementary methods) of the discordant predictions, i.e. the hits that are only predicted by either PolyA-miner or DPU. High clustering stability is observed for PolyA-miner only predictions (Figure 3F) with a median stability score of 0.73 over DPU only predictions (of 0.51). Obvious differential APA patterns (Figure 3C, Supplementary Figure S5), enrichment of UGUA motif (Figure 3D, Supplementary Figure S6), and high clustering stability (Figure 3F) strongly substantiate the validity of PolyA-miner novel predictions.

### PolyA-miner competently reveal non-distal or non-proximal APA dynamics

To understand the methodological advantages of PolyA-miner, we examined the genes that are consistent between PolyA-miner and DPU and respective novel predictions. The APA shift from the most distal to the most proximal site in the gene *TUSC2* (Figure 3G) is detected by both PolyA-miner and DPU. The APA proportion matrix also confirms the same (Figure 3I). With just one source-sink (distal-proximal) pair, such changes in general are simpler to identify. However, in the gene *UBTF* (Figure 3H) the alternative polyadenylation switch from the most distal polyadenylation site (PA) 3 to the intermediate sites (PA2) is predicted only by PolyA-miner. The vector projection metric of PolyA-miner can effectively reflect changes at all APA sites as described in the methods section. On the other hand, the DPU approach computes only distal to proximal usage, ignoring all other non-proximal to non-distal changes. The APA proportion matrix also show decrease in PA3 and increase in PA2 proportions in *NUDT21* KD samples (Figure 3J). This conclusively support PolyA-miner prediction. A significant fraction, about 33% of the genes with APA changes in *NUDT21* KD PAC-Seq data are not distal to proximal changes. This strongly demonstrates the merit of PolyA-miner in identifying broader APA dynamics. The DPU method did predict some candidate genes that PolyA-miner did not. For example, it predicted differential APA usage in the gene *GNS* whereas PolyA-miner did not. However, the sample variability and low consensus clustering stability score (0.47) makes it less likely to be true. One of the *NUDT21* KD sample is as high as controls at the most distal site and is significantly distant from the other two replicates (Supplementary Figure S7a and S7b; high variable sites are annotated with stars). PolyA-miner penalizes such debatable predictions with the intra and inter clustering frequency-based beta statistic.

### PolyA-miner 3′UTR score accurately reflects gene level 3′UTR shortening

**Figure 4.**
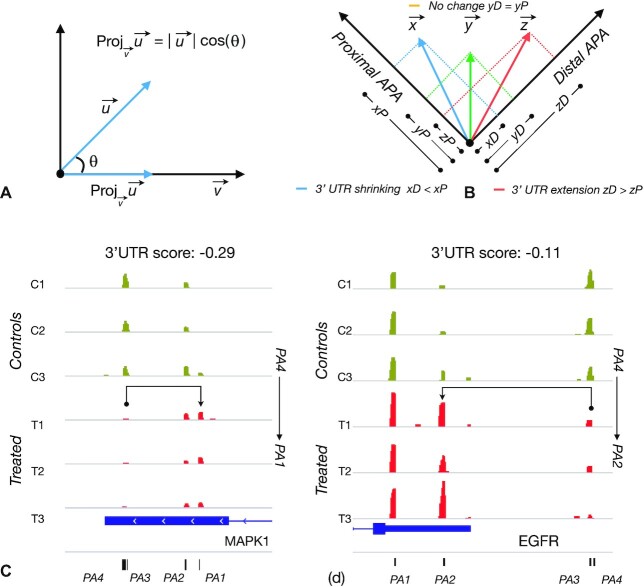
Accurate assessment of 3′UTR shortening/elongation using vector projections: (**A**) Projection of vector *u* on *v*. (**B**) Illustration of 3′UTR changes. Shown in blue is 3′UTR shrinking with control distal projection is greater than that of treated. Conversely, shown in red is 3′UTR elongation with treated distal projection is greater than that of control. Shown in green is when control distal projection is equal to that of treated indicating no change in 3′UTR. (**C**) APA switch from most distal (PA4) to most proximal site (PA1) in MAPK1 and (**D**) APA switch from switch PA4 to PA2 in EGFR.

Typical differential analyses rank respective hits by mere fold change, but this is not appropriate for APA changes. For example, the gene *MAPK1* the distal site is drifted to the most proximal sites (Figure 4C). The proximal site PA1 is increased by 4.5-fold in *NUDT21* KD samples. On the other hand, in the gene EGFR distal sites are shifted to an intermediate site with 4.7-fold increase (Figure 4D). Just by the magnitude of fold change at PA2 *EGFR* ranks higher to MAPK1. However, the proportion of transcripts with the most proximal PA site (shortest 3UTR) is high in *MAPK1*. Shorter the 3′UTR a transcript is more likely to lose miRNA binding sites and less likely to be down regulated. With the shortest dominant isoform *MAPK1* (Figure 4C) is more likely to have stronger downstream effect when compared to *EGFR* (Figure 4D) with second shortest dominant isoform. Thus, in the context of 3′UTR shortening/elongation, the ranking metric should reflect both magnitude and position of APA (3′UTR length) changes. Using vector projections (described in methods) PolyA-miner can account for both the position and magnitude of change. PolyA-miner scoring aptly suggests an overall greater shortening effect in *MAPK1* (−0.29) over *EGFR* (−0.11). This ranking is critical for any downstream analysis that takes rank as their input, such as Gene Set Enrichment Analysis (GSEA).

### PolyA-miner predictions translate to biological insights

Phenotype analysis of PolyA-miner predicted genes with 3′UTR shortening showed enrichment for intellectual disability, neurodevelopmental delays and other neuropsychiatric phenotypes (Figure 3E and Supplementary Table S3). These align with the recent advances in *NUDT21* biology (39,40). On the other hand, phenotype enrichment of DPU predictions is limited (Supplementary Figure S7c, Table S4). Furthermore, in *de-novo* APA site detection mode, PolyA-miner identified 3074 novel polyadenylation sites that were otherwise not reported in PolyA_DB (Supplementary Table S5).

### Widespread longer 3′UTR isoforms are observed in MAQC human brain PolyA-seq data

**Figure 5.**
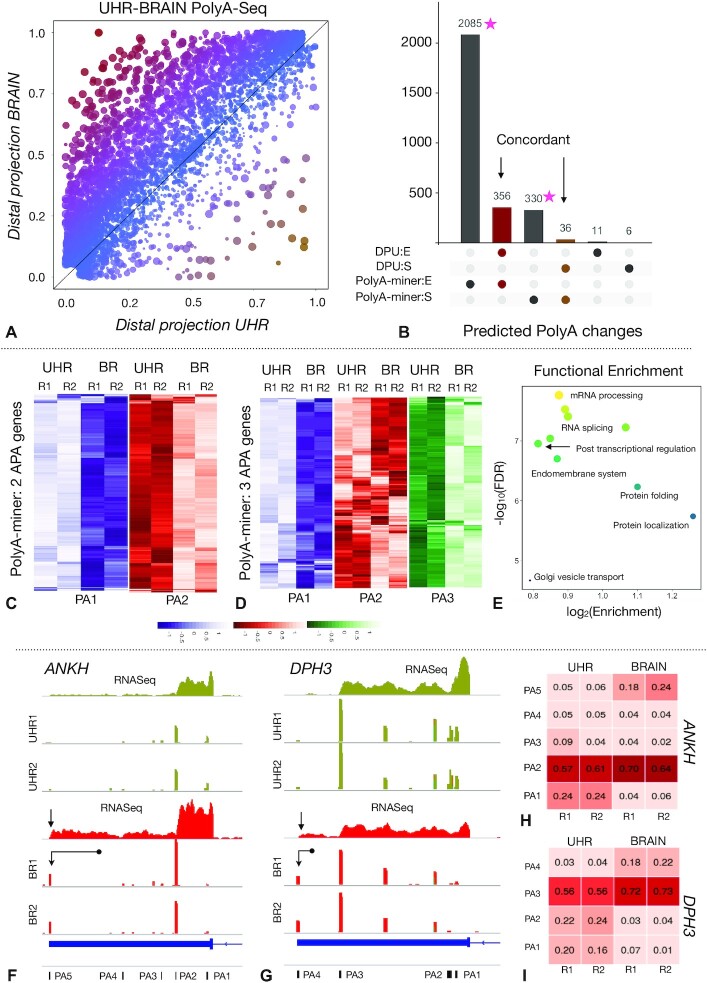
(**A**) PolyA-miner reveals extensive 3′UTR elongation in MAQC brain and UHR PolyA-seq data: Size of the marker indicate statistical significance. Color gradient (red to orange) indicate 3′UTR length long to short. (**B**) Overlap and discordance of PolyA-miner and DPU predictions. (**C**) APA heatmap of genes with two polyadenylations and (**D**) 3 polyadenylations sites. (**E**) Functional analysis of PolyA-miner prediction (GO terms with FDR ≤ 0.05). (**F** and **G**) Tracks showing 3′UTR elongations identified by both PolyA-miner and DPU, and only. Distal APA sites (elongation) is annotated with arrows and corresponding RNASeq tracks are also shown. (**H** and **I**) APA proportion matrices of the genes shown in F and G respectively.

To demonstrate the protocol independent usability of PolyA-miner, we next evaluated it with MAQC Universal Human Reference (UHR) and human brain PolyA-seq datasets (3). PolyA-seq is one of the first generation 3′ sequencing protocols and requires complex polyA enrichment, sample preparation and purification steps (12). Data was obtained from the GEO database (GSM747473-76). Similar to *NUDT21* KD PAC-Seq data, ∼94% of the putative APA sites are filtered out by mispriming and other de-noising filters (Figure 2c). This reinforces our conclusion about the magnitude of internal priming events and noise in the current 3′Sequencing protocols. In *de-novo* APA site detection mode, PolyA-miner identified 21338 novel polyadenylation sites (Supplementary Table S10). Predominantly distal polyA sites (longer 3′UTR isoforms) are found in human brain, i.e. APA vector projections are more distal in brain when compared to that of UHR (Figure 5A). This observation is consistent with the literature (33).

PolyA-miner identified 2441 3′UTR elongated and 366 shortened genes (Supplementary Table S6). The DPU approach identified 367 3′UTR elongation and 42 3′UTR shrinking events (Supplementary Table S7), out of which 356 and 36 events are also detected by PolyA-miner (Figure 5B). On the other hand, PolyA-miner predicted 2085 and 330 elongations and shortening events that are not detected by DPU. APA heatmap patterns illustrated in Figure 5C, D and Supplementary Figure S8 conclusively validate PolyA-miner novel predictions. PolyA-miner elongation predictions are enriched for RNA splicing, mRNA processing, post translational regulation, (Figure 5E and Supplementary Table S8) which aligns with the high transcriptional and splicing diversity in the brain (41). Functional insights from DPU predictions (Supplementary Figure S9c and Table S9) are limited when compared to that of PolyA-miner predictions.

Both PolyA-miner and DPU predicted 3′UTR elongation in the gene *ANKH* (Figure 5f). APA proportion matrix (Figure 5H) and standard RNASeq of samples obtained from Sequencing Quality Control (SEQC) project also suggest dominant distal polyadenylation site in brain (annotated by an arrow in Figure 5F). However, only PolyA-miner predicted 3′UTR elongation in the gene *DPH3* (Figure 5G). Both regular RNASeq tracks (Figure 5G) and APA proportion matrix (Figure 5I) validate PolyA-miner predictions. There is an increase in proportion of the distal sites PA4 and PA3 in brain samples. On the other hand, only DPU predicted 3′UTR elongation in the gene *RIOK1* (Supplementary Figure S9a). APA proportions (reads) from both proximal and distal sites are redistributed to the intermediate site (Supplementary Figure S9b) making this a debatable prediction. It essentially boils down to change magnitude vs APA site position (length). A detailed experimental investigation is necessary to establish an agreement between the APA position (length) and the magnitude of change to assess the downstream effect.

## CONCLUSION

PolyA-miner is the first differential alternative polyadenylation usage tool that is specifically designed for 3′Seq data. We demonstrated the importance of extensive filtering in pre-processing 3′Seq data. Further, our iterative consensus NMF makes the analysis less susceptible to intra sample variation. Most importantly, using vector projections, PolyA-miner can account for all APA changes including non-proximal to non-distal changes and can distinguish the most distal to most proximal changes from most distal to intermediate site changes irrespective of absolute change magnitude. This sensitivity is extremely important to thoroughly estimate of the true breadth of 3′UTR shortening and elongation. Evaluation on both the first generation MAQC brain and UHR PolyA-seq data, and recent Glioblastoma cell line PAC-seq (3′Seq) data strongly supports the value and protocol independent applicability of PolyA-miner. We demonstrated a substantial increase in both the number of dynamic APA events detected and novel APA sites using PolyA-miner. With the emerging importance of alternative polyadenylation in understanding development and diseases, PolyA-miner can significantly improve data analysis and help decode the missing pieces of underlying alternative polyadenylation dynamics.

## DATA AVAILABILITY

PolyA-miner is implemented in Python and the source code is freely available at http://www.liuzlab.org/PolyA-miner/. The PAC-seq data are available in the NCBI Gene Expression Omnibus (GEO), accession number: GSE147661.

## Supplementary Material

gkaa398_Supplemental_Files
