# Supplementary material for: PolyA-miner: accurate assessment of differential alternative poly-adenylation from 3′Seq data using vector projections and non-negative matrix factorization
Source: Nucleic Acids Res. 2020 May 28;48(12):e69. doi: 10.1093/nar/gkaa398 (PMC7337927; doi:10.1093/nar/gkaa398)
Supplement: gkaa398_Supplemental_Files [file gkaa398_supplemental_files.zip › PolyA-miner_SupplementaryFigures.pdf]

Figure S1

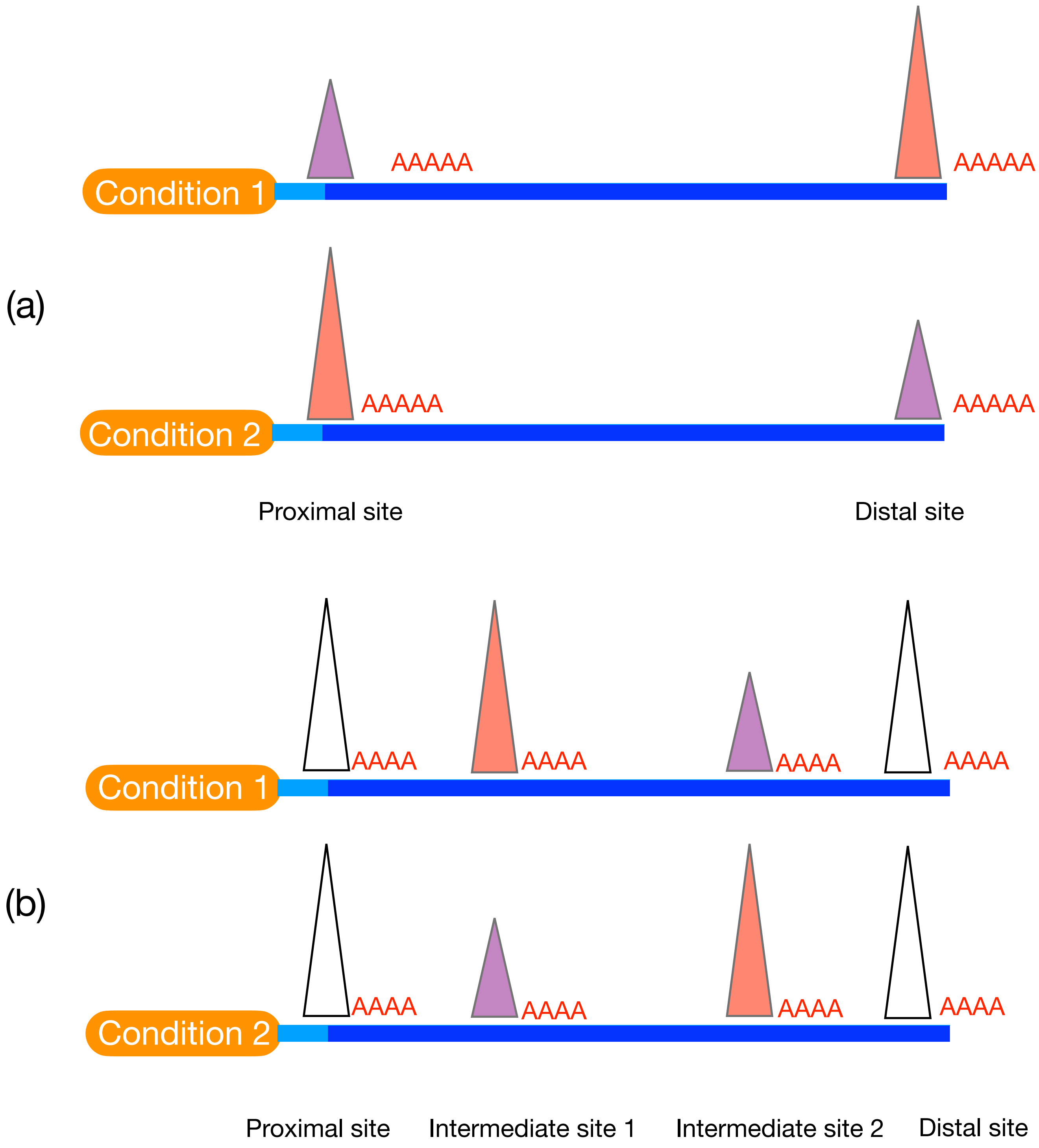

Condition 1
AAAAA
AAAAA
(a)
Condition 2
AAAAA
AAAAA
Proximal site
Distal site
Condition 1
AAAA
AAAA
AAAA
AAAA
(b)
Condition 2
AAAA
AAAA
AAAA
AAAA
Proximal site
Intermediate site 1
Intermediate site 2
Distal site

Figure S2

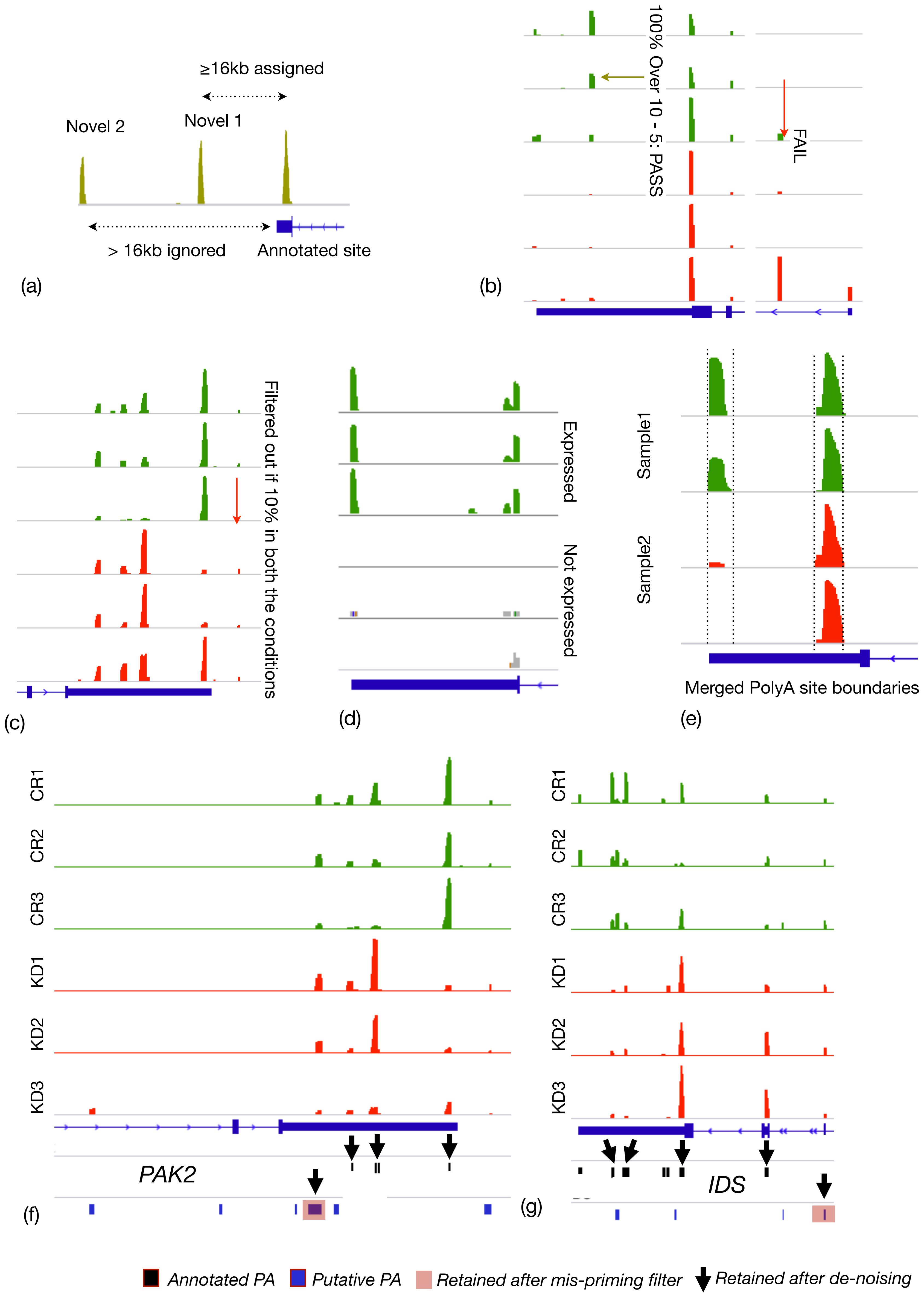

≥16kb assigned
Novel 2
Novel 1
> 16kb ignored
Annotated site
(a)
100% Over 10 - 5: PASS
FAIL
(b)
Filtered out if 10% in both the conditions
(c)
Expressed
Not expressed
(d)
Sample1
Sample2
Merged PolyA site boundaries
(e)
CR1
CR2
CR3
KD1
KD2
KD3
PAK2
(f)
IDS
(g)
Annotated PA
Putative PA
Retained after mis-priming filter
Retained after de-noising

Figure S3

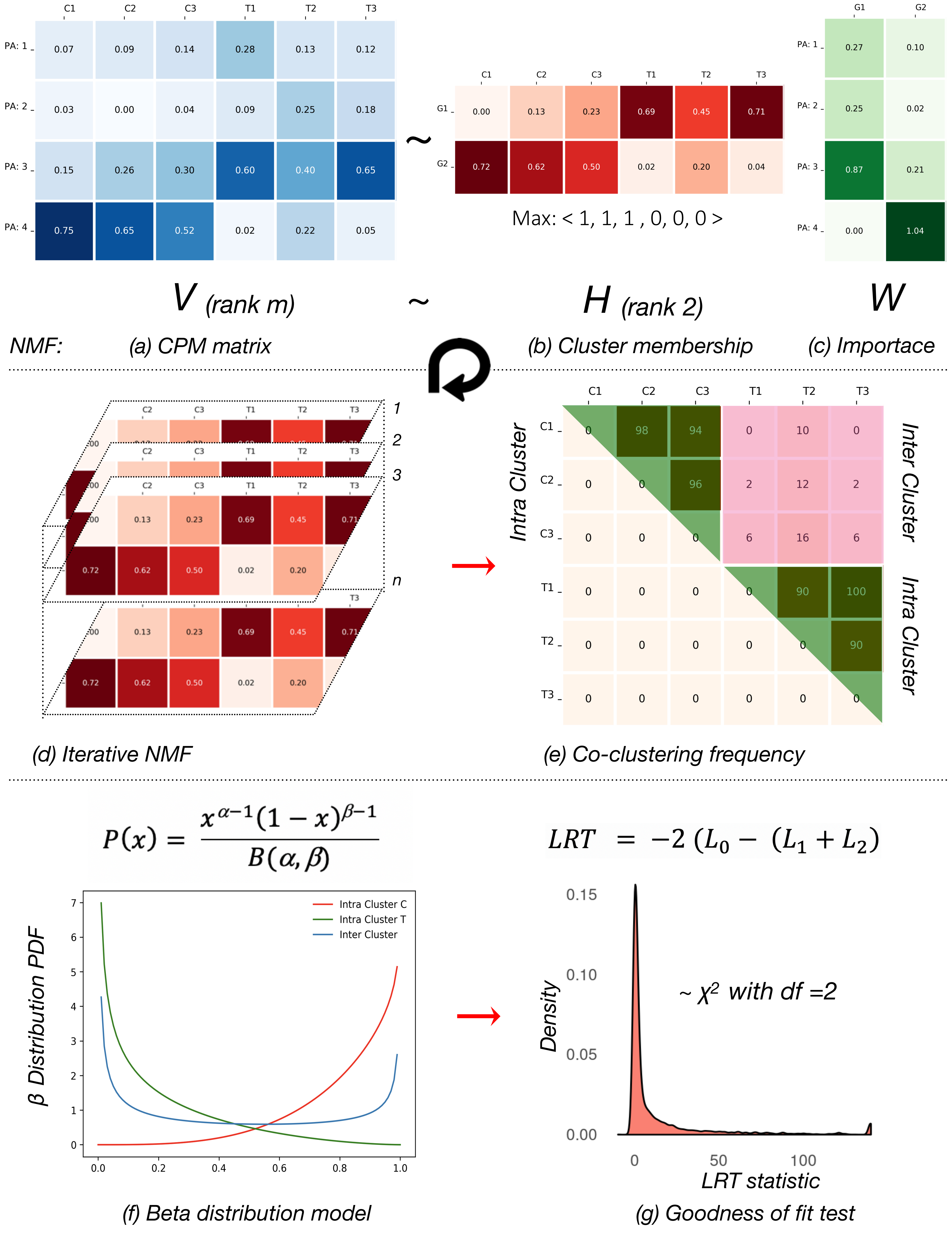

$$P(x) = \frac{x^{\alpha-1}(1-x)^{\beta-1}}{B(\alpha,\beta)}$$

$$LRT = -2\,(L_0 - (L_1 + L_2))$$

Figure S4

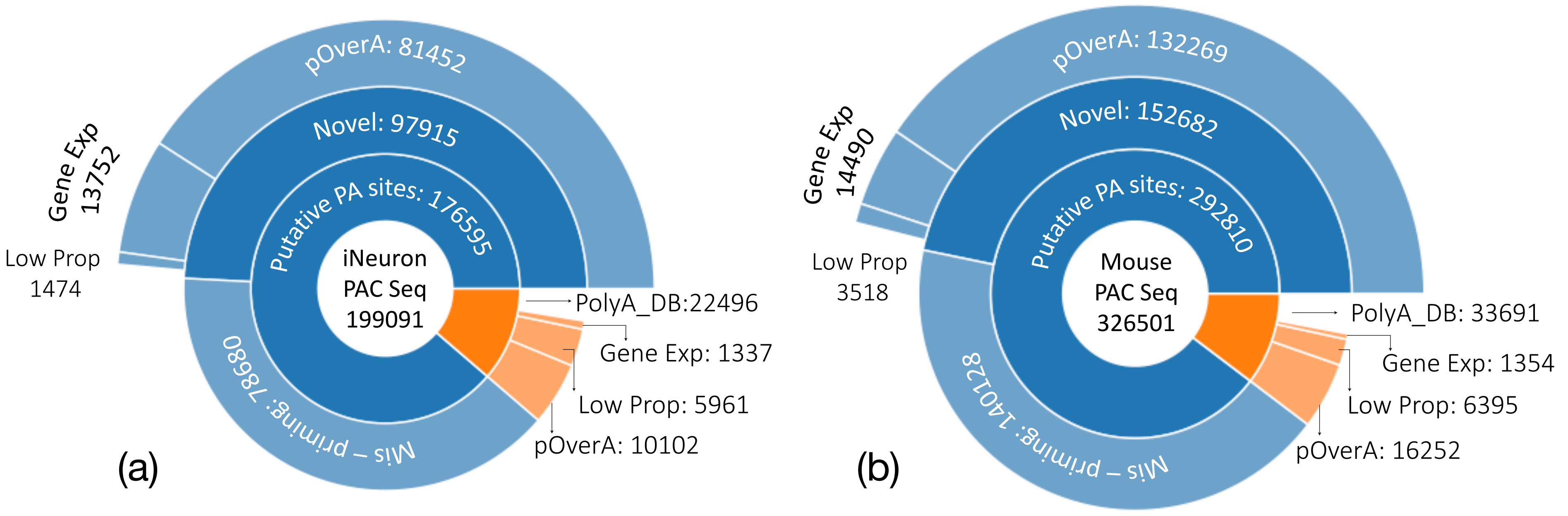
pOverA: 81452
Novel: 97915
Gene Exp
13752
Low Prop
1474
Putative PA sites: 176595
iNeuron
PAC Seq
199091
PolyA_DB:22496
Gene Exp: 1337
Low Prop: 5961
pOverA: 10102
Mis – priming: 78680
(a)
pOverA: 132269
Novel: 152682
Gene Exp
14490
Low Prop
3518
Putative PA sites: 292810
Mouse
PAC Seq
326501
PolyA_DB: 33691
Gene Exp: 1354
Low Prop: 6395
pOverA: 16252
Mis – priming: 140128
(b)

Figure S5

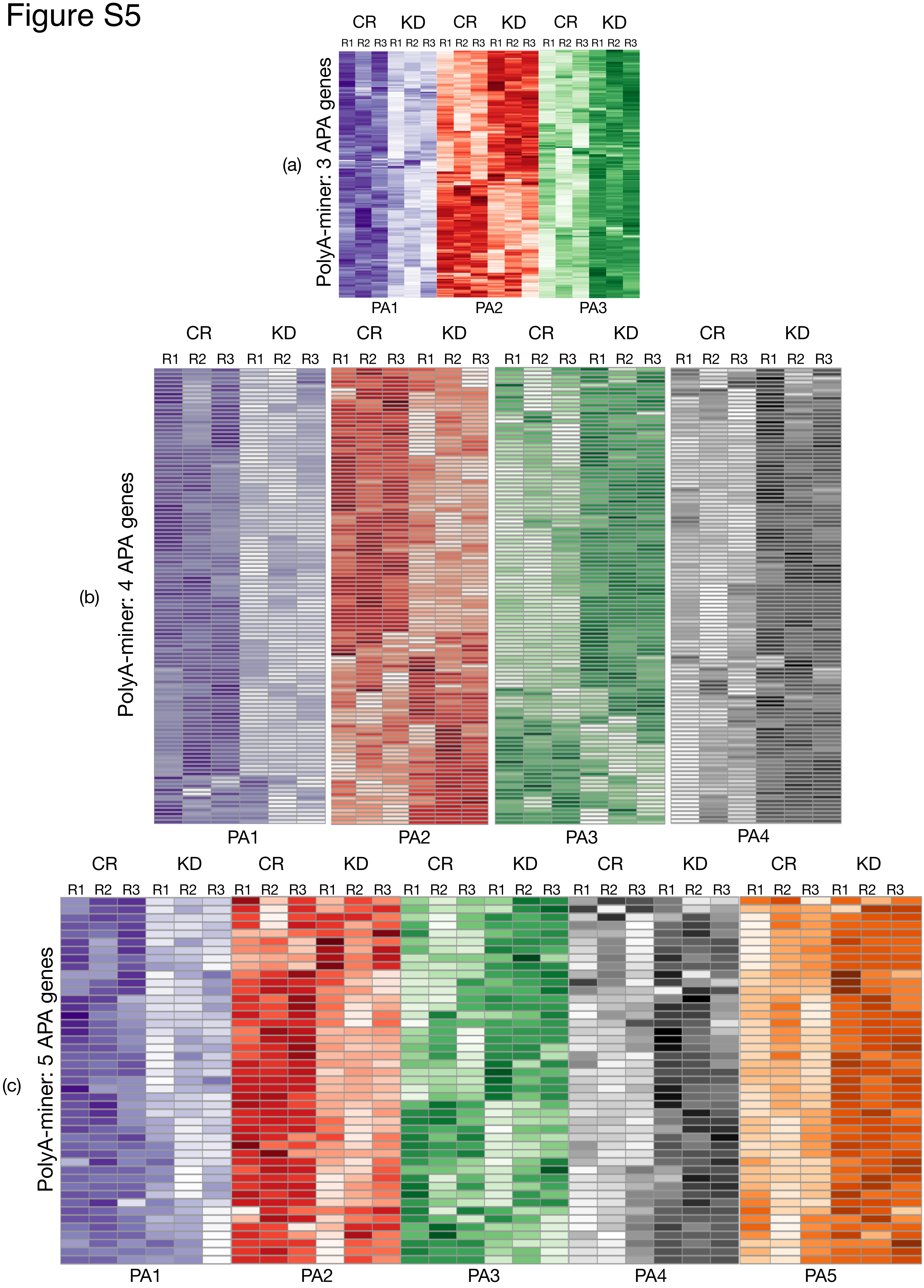

(a)
PolyA-miner: 3 APA genes
CR KD CR KD CR KD
R1 R2 R3 R1 R2 R3 R1 R2 R3 R1 R2 R3 R1 R2 R3 R1 R2 R3
PA1 PA2 PA3
(b)
PolyA-miner: 4 APA genes
CR KD CR KD CR KD CR KD
R1 R2 R3 R1 R2 R3 R1 R2 R3 R1 R2 R3 R1 R2 R3 R1 R2 R3 R1 R2 R3 R1 R2 R3
PA1 PA2 PA3 PA4
(c)
PolyA-miner: 5 APA genes
CR KD CR KD CR KD CR KD CR KD
R1 R2 R3 R1 R2 R3 R1 R2 R3 R1 R2 R3 R1 R2 R3 R1 R2 R3 R1 R2 R3 R1 R2 R3 R1 R2 R3 R1 R2 R3
PA1 PA2 PA3 PA4 PA5

Figure S6

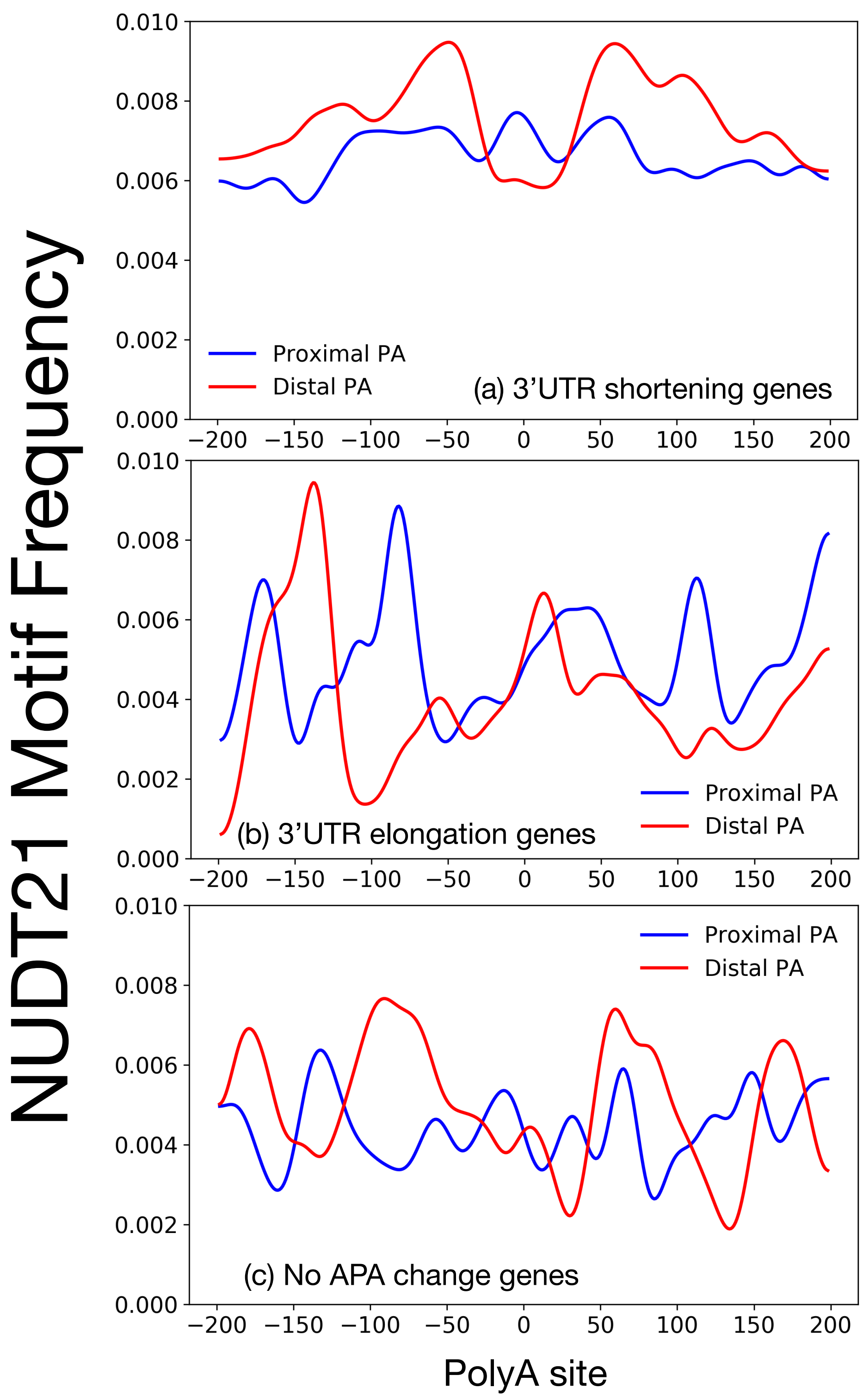

NUDT21 Motif Frequency
0.010
0.008
0.006
0.004
0.002
0.000
Proximal PA
Distal PA
(a) 3'UTR shortening genes
−200 −150 −100 −50 0 50 100 150 200
(b) 3'UTR elongation genes
(c) No APA change genes
PolyA site

Figure S7

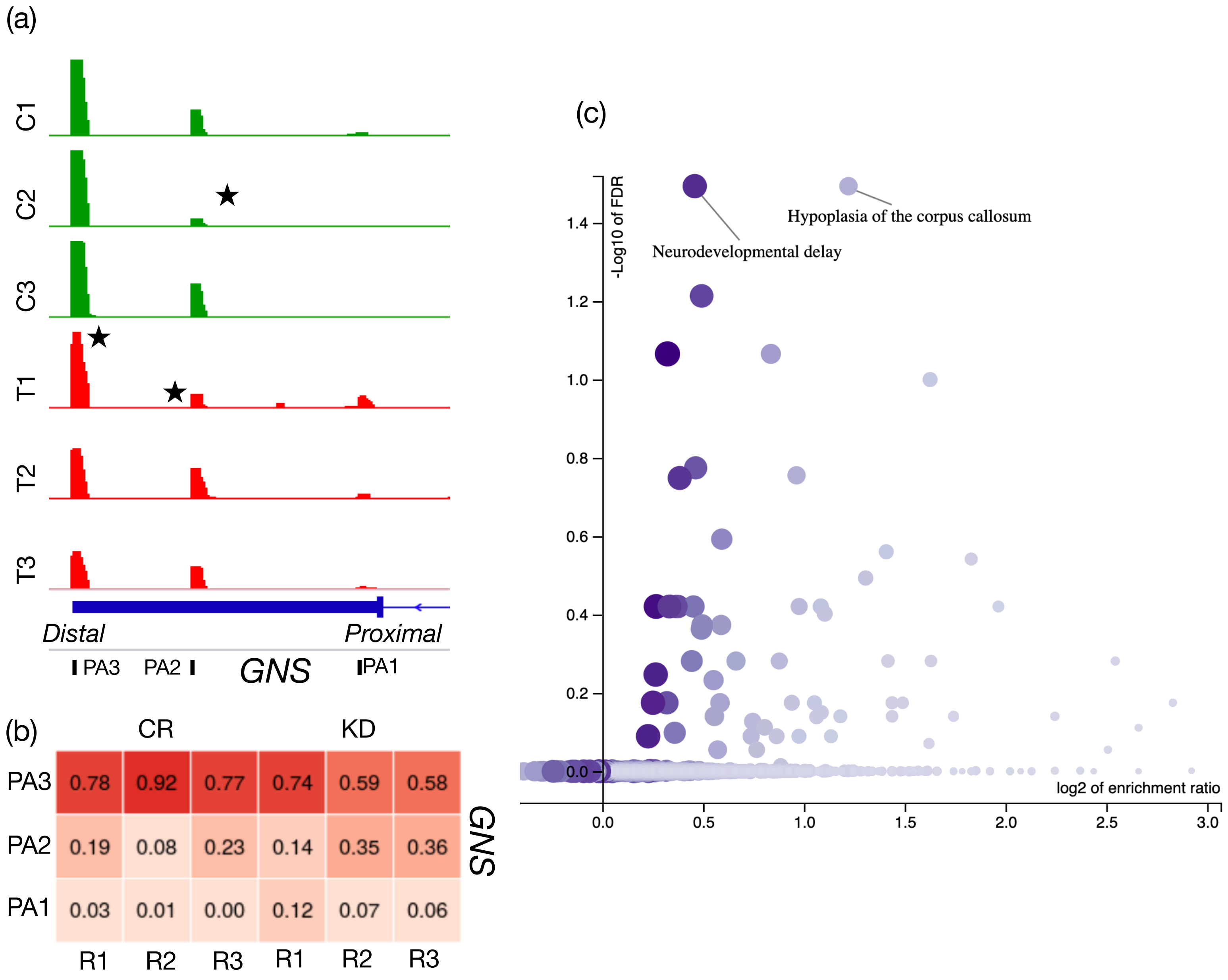

(a)
C1
C2
C3
T1
T2
T3
Distal
Proximal
PA3
PA2
GNS
PA1
(b)
CR
KD
PA3 0.78 0.92 0.77 0.74 0.59 0.58
PA2 0.19 0.08 0.23 0.14 0.35 0.36
PA1 0.03 0.01 0.00 0.12 0.07 0.06
R1 R2 R3 R1 R2 R3
GNS
(c)
-Log10 of FDR
Hypoplasia of the corpus callosum
Neurodevelopmental delay
log2 of enrichment ratio
0.0 0.2 0.4 0.6 0.8 1.0 1.2 1.4
0.0 0.5 1.0 1.5 2.0 2.5 3.0

Figure S8

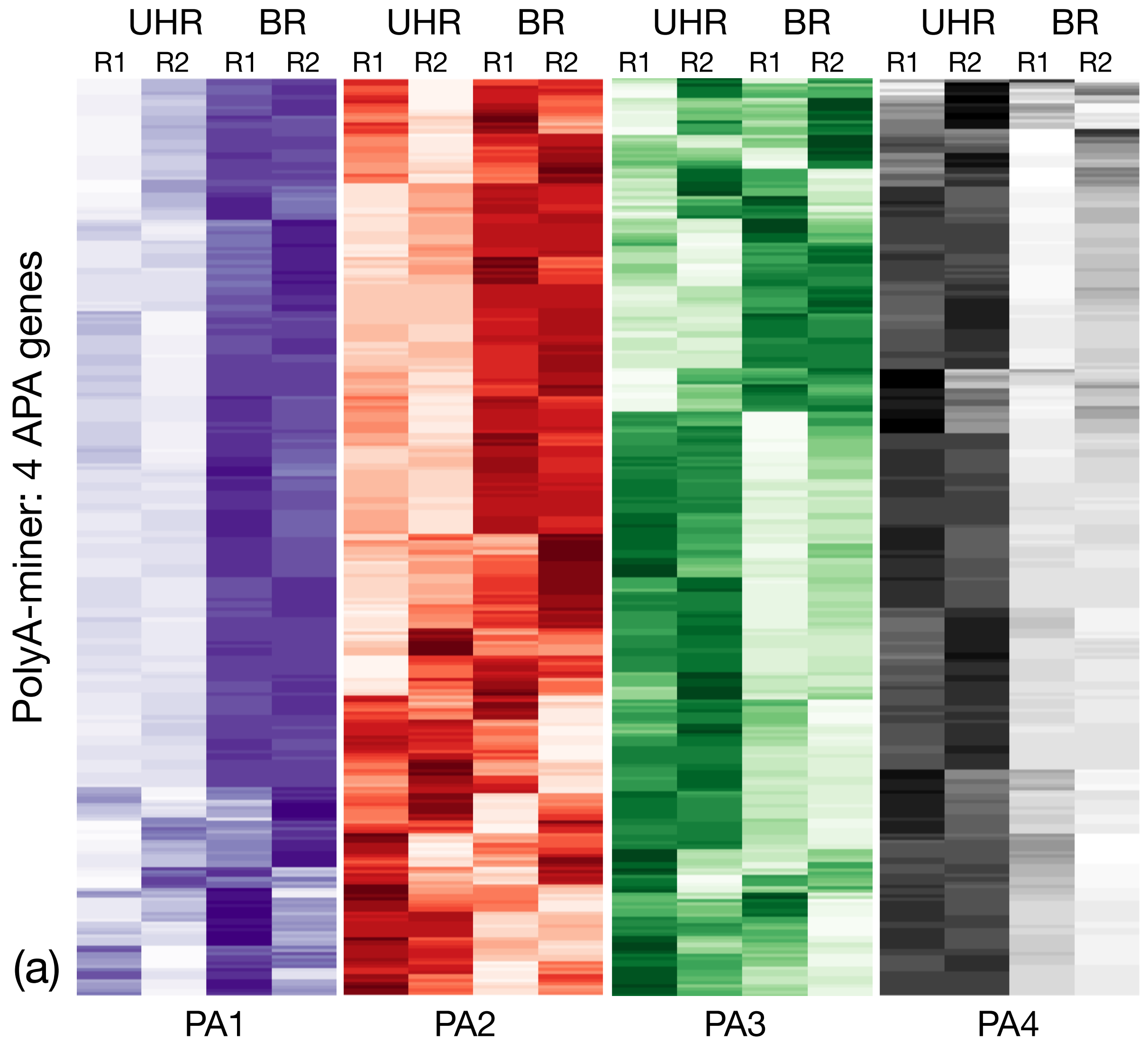

UHR BR UHR BR UHR BR UHR BR
R1 R2 R1 R2 R1 R2 R1 R2 R1 R2 R1 R2 R1 R2 R1 R2
PolyA-miner: 4 APA genes
(a)
PA1 PA2 PA3 PA4

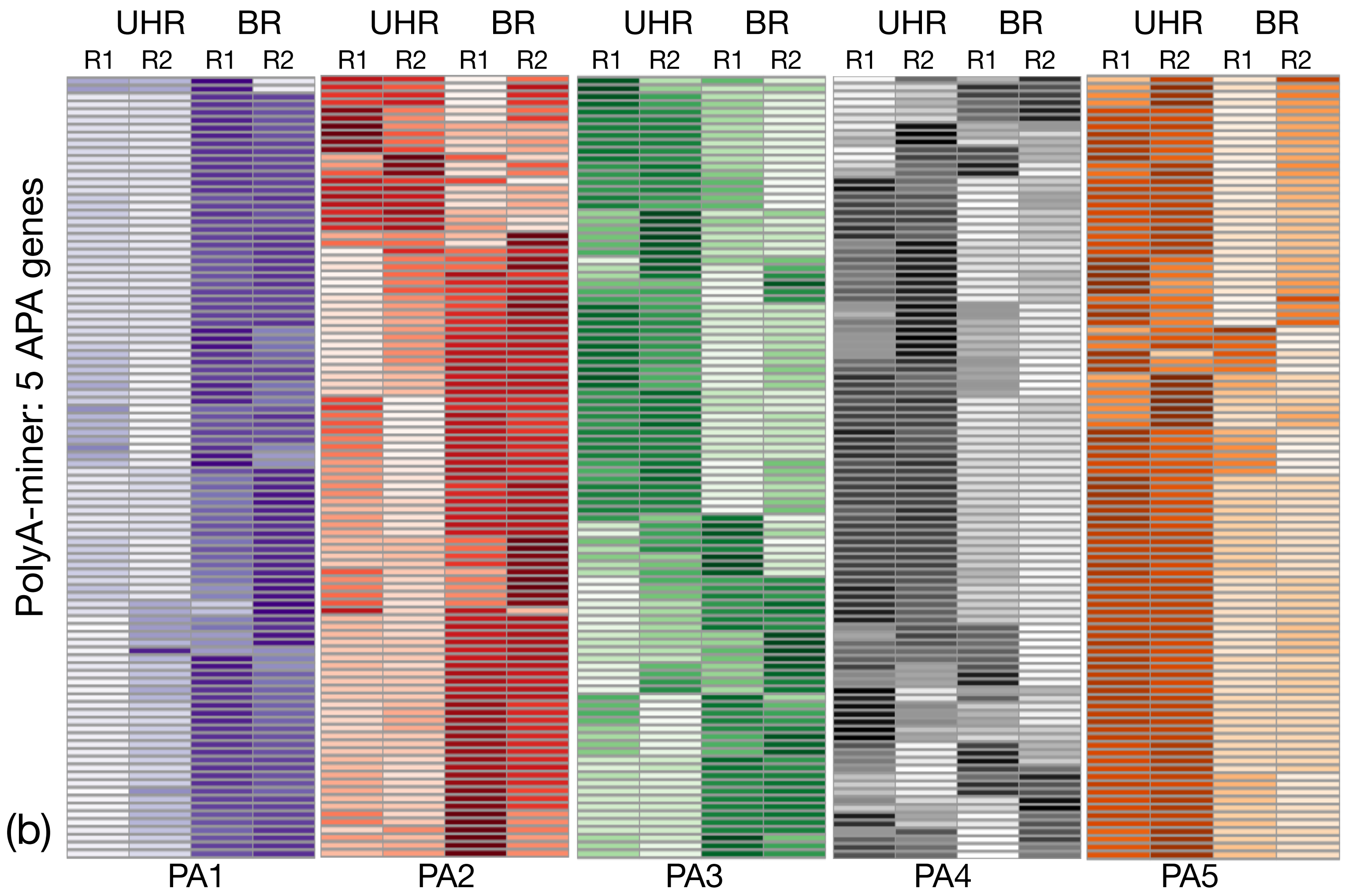

UHR BR UHR BR UHR BR UHR BR UHR BR
R1 R2 R1 R2 R1 R2 R1 R2 R1 R2 R1 R2 R1 R2 R1 R2 R1 R2 R1 R2
PolyA-miner: 5 APA genes
(b)
PA1 PA2 PA3 PA4 PA5

Figure S9

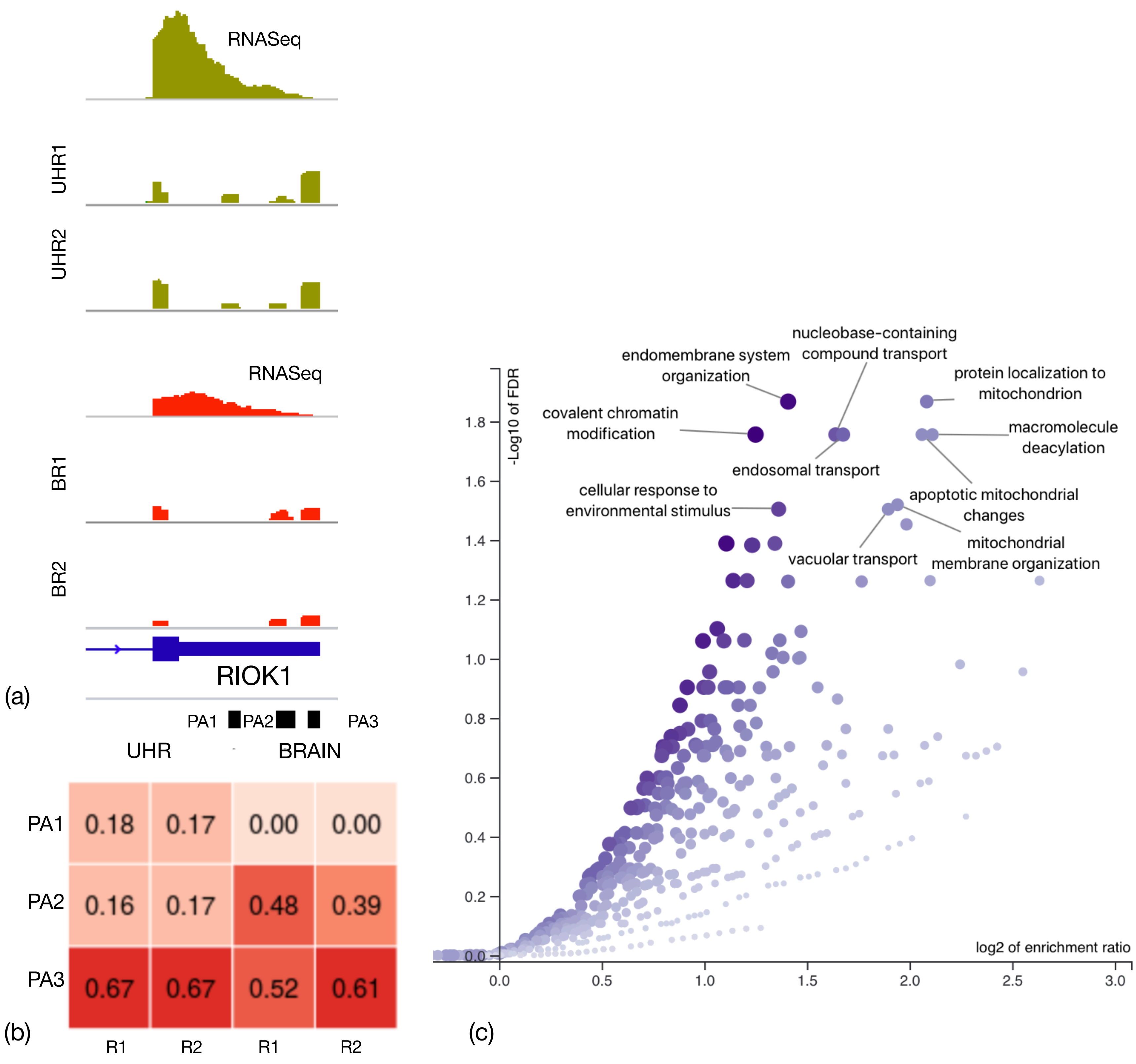
RNASeq
UHR1
UHR2
RNASeq
BR1
BR2
RIOK1
(a)
PA1
PA2
PA3
UHR
BRAIN
PA1 0.18 0.17 0.00 0.00
PA2 0.16 0.17 0.48 0.39
PA3 0.67 0.67 0.52 0.61
R1 R2 R1 R2
(b)
nucleobase-containing compound transport
endomembrane system organization
protein localization to mitochondrion
covalent chromatin modification
macromolecule deacylation
endosomal transport
cellular response to environmental stimulus
apoptotic mitochondrial changes
mitochondrial membrane organization
vacuolar transport
-Log10 of FDR
1.8
1.6
1.4
1.2
1.0
0.8
0.6
0.4
0.2
0.0
log2 of enrichment ratio
0.0
0.5
1.0
1.5
2.0
2.5
3.0
(c)
