# Supplementary material for: PolyA-miner: accurate assessment of differential alternative poly-adenylation from 3′Seq data using vector projections and non-negative matrix factorization
Source: Nucleic Acids Res. 2020 May 28;48(12):e69. doi: 10.1093/nar/gkaa398 (PMC7337927; doi:10.1093/nar/gkaa398)
Supplement: gkaa398_Supplemental_Files [file gkaa398_supplemental_files.zip › Supplemental_Figure_Table_Legends.docx]

**Supplemental Figure Legends**

**Figure S1:** Representative illustration of (a) Distal to proximal APA change. (b) Non-distal to non-proximal APA change.

**Figure S2:** PolyA-miner APA filers: (a) APA to gene annotation threshold. (b) pOverA filter: Pointed in green and red are the representative passed and failed APA sites respectively. (c) Proportion filter: Pointed in red is the filtered representative low proportion site (< 10%). (d) Gene expression filter to control for non-expressed genes. (e) Merging overlapping polyadenylation sites: Dotted lines indicate respective merged polyadenylation site boundaries. (f and g) Illustration of mispriming and other noise levels in the genes PAK2 and IDS from *NUDT21* KD PAC-Seq data respectively. Shown in black are annotated sites from PolyA_DB. Putative sites are shown in blue. Masked in grey are the sites retained after mispriming filter and Marked with arrow are the sites retained after de-noising filters.

**Figure S3:** Illustration of iterative NMF flow on the gene PBX3 in Glioblastoma cell line *NUDT21* KD dataset: (a) NMF input -normalized APA proportion matrix. (b) *H* matrix indicating cluster membership. (c) *W* matrix indicating respective individual APA weights. (d) Multiple *H* matrices from iterative NMF runs. (e) Co-clustering frequency matrix summarizing sample intra and inter clustering frequencies over the multiple NMF runs. (f) Estimating beta distribution from co-clustering frequency matrix and (g) Likely hood ratio test over a null model.

**Figure S4:** Distribution of the filtered putative and annotated APA sites by respective de-noising filters in (a) Control human neuron PAC-Seq data. (b) Wild type mouse hippocampus PAC-Seq data.

**Figure S5:** Heatmaps of PolyA-miner only predictions showing differential APA usage: (a) Genes with 3 APA sites. (b) Genes with 4 APA sites. (c) Genes with 5 APA sites in *NUDT21* KD PAC-Seq data.

**Figure S6:** NUDT21 motif frequency in the genes with (a) Predicted 3′UTR shortening. (b) Predicted 3′UTR elongation. (c) No significant APA changes.

**Figure S7:** (a) Tracks showing 3′UTR shortening identified by DPU but missed by PolyA-miner in *NUDT21* KD PAC-Seq data. (b) Corresponding APA proportion matrix showing intra sample variability. (c) Phenotype enrichment of the as 3′UTR shortening genes predicted by DPU.

**Figure S8:** Heatmaps of PolyA-miner only predictions showing differential APA usage: (a) Genes with 4 APA sites and (b) 5 APA sites in MAQC brain and UHR PolyA-seq data.

**Figure S9:** (a) Tracks showing 3′UTR elongation identified by DPU but missed by PolyA-miner in MAQC brain and UHR PolyA-seq data. (b) Corresponding APA proportion matrix showing intra sample variability. (c) Functional enrichment of the as 3′UTR shortening genes predicted by DPU.

**Supplemental Tables**

**Table S1:** 3′ UTR changes in NUDT21 KD data set predicted using PolyA-miner.

**Table S2:** 3′ UTR changes in NUDT21 KD data set predicted using DPU approach.

**Table S3:** Phenotype enrichment table of 3′UTR shortening genes in NUDT21 KD data set predicted by PolyA-miner.

**Table S4:** Phenotype enrichment table of 3′UTR shortening genes in NUDT21 KD data set predicted by DPU approach.

**Table S5:** Novel polyadenylation sites detected in NUDT21 KD data set using PolyA-miner.

**Table S6:** 3′ UTR changes in MAQC UHR-brain data set predicted using PolyA-miner.

**Table S7:** 3′ UTR changes in MAQC UHR-brain data set predicted using DPU approach.

**Table S8:** Functional enrichment table of 3′UTR lengthening genes in MAQC UHR-brain data set predicted by PolyA-miner.

**Table S9:** Functional enrichment table of 3′UTR lengthening genes in MAQC UHR-brain data set predicted by DPU approach.

**Table S10:** Novel polyadenylation sites detected in MAQC UHR-brain data set using PolyA-miner.
