# Supplementary material for: PolyA-miner: accurate assessment of differential alternative poly-adenylation from 3′Seq data using vector projections and non-negative matrix factorization
Source: Nucleic Acids Res. 2020 May 28;48(12):e69. doi: 10.1093/nar/gkaa398 (PMC7337927; doi:10.1093/nar/gkaa398)
Supplement: gkaa398_Supplemental_Files [file gkaa398_supplemental_files.zip › Supplementary_methods_Revision.docx]

**SUPPLEMENTARY METHODS**

**Distal to Proximal Usage (DPU)**

Raw data (fastq files) were process the same way as described in the main text. Similar to PolyA-miner consistent mispriming and other denoising filters are also used in distal to proximal usage (DPU) analysis. Once the APA matrix is generated distal to proximal usage is computed using the most distal and the most proximal polyA sites as the ratio of distal read counts to the total reads (distal + proximal).

$$DPU= \frac{DPA r}{(DPA r+PPA r)}$$

Where DPAr is the number of reads mapped to the distal polyA site and PPAr is the number of reads mapped to the proximal polyA site. DPU essentially measures the enrichment of distal polyA site. Genes with dominant distal site have higher DPU values and vice versa.

Sample-wise DPU values are compared between the control and the treatment groups. Genes with less distal to proximal usage in treatment group are predicted as 3’URT shortening and vice-versa. A t-test is performed to evaluate the statistical significance.

**Clustering Stability score**

Clustering stability score of a gene is computed as the ratio of intra-group clustering frequency to intra and inter group clustering frequency as bellow:

$${CS}_{G}=\frac{\sum_{g=1}^{2} {CF}_{i,j|g}}{\left( \sum_{g=1}^{2} {CF}_{i,j|g}+\sum_{g=}^{n} {CF}_{ig1,jg2} \right)}$$

Where ${CS}_{G}$ is the clustering stability of the gene G. ${CF}_{i,j|g}$ is the (intra-group) co-clustering frequency of sample *i* and *j* of group *g*. ${CF}_{ig1,jg2}$ is the (inter-group) co-clustering frequency of sample *i* from group *g1* and *j* in group *g2*.

**Phenotype and functional enrichment analysis**

Phenotype and Functional enrichment analyses were performed on gene lists of interest using WebGestalt (WEB-based GEne SeT AnaLysis Toolkit) (1) with an FDR cutoff of 0.05. Human phenotype ontologies were used for phenotype enrichment and Gene Ontology (Biological process terms) were used for functional enrichment. Minimum number of genes per category was set to 5.

**Mouse husbandry and handling**

The Baylor College of Medicine Institutional Animal Care and Use Committee approved all mouse care and manipulation (IACUC, protocol AN-1013). We housed wild-type, C57BL/6J mice in an AAALAS-certified level three facility on a 14-hour light cycle with ad libitum access to standard chow and water.

**Human embryonic stem cell (hESC)-derived neuron culture**

We used WA09 (H9; RRID:CVCL_9773, WiCell) female embryonic stem cells (ESCs) to generate human neurons as previously described (2). We confirmed their identity by STR analysis and verified that they were free of mycoplasma. We differentiated neural progenitors into human neurons over three weeks, changing the media every 3 days. Afterwards, we passaged the neurons with trypsin. Three days after passaging, as a control for another experiment, we infected the neurons with lentiviruses containing pGIPZ non-silencing shRNA clone RHS4348 (Dharmacon) at a multiplicity of infection of ten. We verified the tropism and infectivity of the virus using the tGFP reporter signal. On day two after infection, we treated the cells with 1 µM 5-Fluoro-2′-deoxyuridine for one day to remove proliferating glia from the culture (3). At day three, we treated the neurons with puromycin (0.75–1.25 g/ml) for six days to select for infected cells. We cultured the cells for 60 days after infection, changing the media three times per week. We then aspirated all the media and washed the cells with PBS before freezing them at -80C for later RNA extraction.

**Poly(A) click-seq**

*RNA extraction*

We extracted RNA from the hippocampi of male 46-week-old wild-type mice, as well as human ESC-derived excitatory neurons in a 12-well plate. We prepared four samples per genotype to allow for loss of one sample. We lysed the tissue with TRIzol Reagent (ThermoFisher Scientific) and immediately transferred the lysate to microfuge tubes for trituration. We then isolated the RNA by chloroform phase separation, precipitation with 2-propanol, washing with 75% ethanol, and eluting in water.

*Library preparation and sequencing*

We prepared sequencing libraries as previously described (4). We reverse transcribed 1 ug of total RNA with the partial P7 adapter (Illumina_4N_21T) and dNTPs with the addition of spiked-in azido-nucleotides (AzVTPs) at 5:1. We click-ligated the p5 adapter (IDT) to the 5′ end of the cDNA with CuAAC. We then amplified the cDNA for 21 cycles with Universal primer and 3′ indexing primer and purified it on a 2% agarose gel by extracting amplicon from 200-300 base pairs. We pooled the libraries and sequenced single-end, 75 base-pair reads on a Nextseq 550 (Illumina).

P7 adapter (Illumina_4N_21T):

GTGACTGGAGTTCAGACGTGTGCTCTTCCGATCTNNNNTTTTTTTTTTTTTTTTTTTTT

P5 adapter (IDT):

5′HexynylNNNNAGATCGGAAGAGCGTCGTGTAGGGAAAGAGTGTAGATCTCGGTGGTCGCCGTATCATT

Universal primer:

AATGATACGGCGACCACCGAG

Example 3′ indexing primer:

CAAGCAGAAGACGGCATACGAGATCGTGATGTGACTGGAGTTCAGACGTGT

**REFERENCES**

1. Liao,Y., Wang,J., Jaehnig,E.J., Shi,Z. and Zhang,B. (2019) WebGestalt 2019: gene set analysis toolkit with revamped UIs and APIs. *Nucleic Acids Research*, 10.1093/nar/gkz401.

2. Jiang,X., Chen,J., Bajić,A., Zhang,C., Song,X., Carroll,S.L., Cai,Z.L., Tang,M., Xue,M., Cheng,N., *et al.* (2017) Quantitative real-time imaging of glutathione. *Nature Communications*, 10.1038/ncomms16087.

3. Hui,C.W., Zhang,Y. and Herrup,K. (2016) Non-Neuronal Cells Are Required to Mediate the Effects of Neuroinflammation: Results from a Neuron-Enriched Culture System. *PLoS ONE*, 10.1371/journal.pone.0147134.

4. Routh,A., Ji,P., Jaworski,E., Xia,Z., Li,W. and Wagner,E.J. (2017) Poly(A)-ClickSeq: Click-chemistry for next-generation 3’-end sequencing without RNA enrichment or fragmentation. *Nucleic Acids Research*, 10.1093/nar/gkx286.
